# Supplementary material for: Revisiting Microbial Diversity in Hypersaline Microbial Mats from Guerrero Negro for a Better Understanding of Methanogenic Archaeal Communities
Source: Microorganisms. 2023 Mar 22;11(3):812. doi: 10.3390/microorganisms11030812 (PMC10059902; doi:10.3390/microorganisms11030812)
Supplement: Supplementary file 1 [file microorganisms-11-00812-s001.zip › Supplementary Figures and Tables.pdf]

Supplementary Materials

Revisiting microbial diversity in hypersaline microbial mats from Guerrero Negro for a better understanding of methanogenic archaeal communities.

García-Maldonado José Q., Latisnere-Barragán Hever, Escobar-Zepeda Alejandra, Cadena Santiago, Ramírez-Arenas Patricia J., Vázquez-Juárez Ricardo, Rojas-Contreras Maurilia and López-Cortés Alejandro.

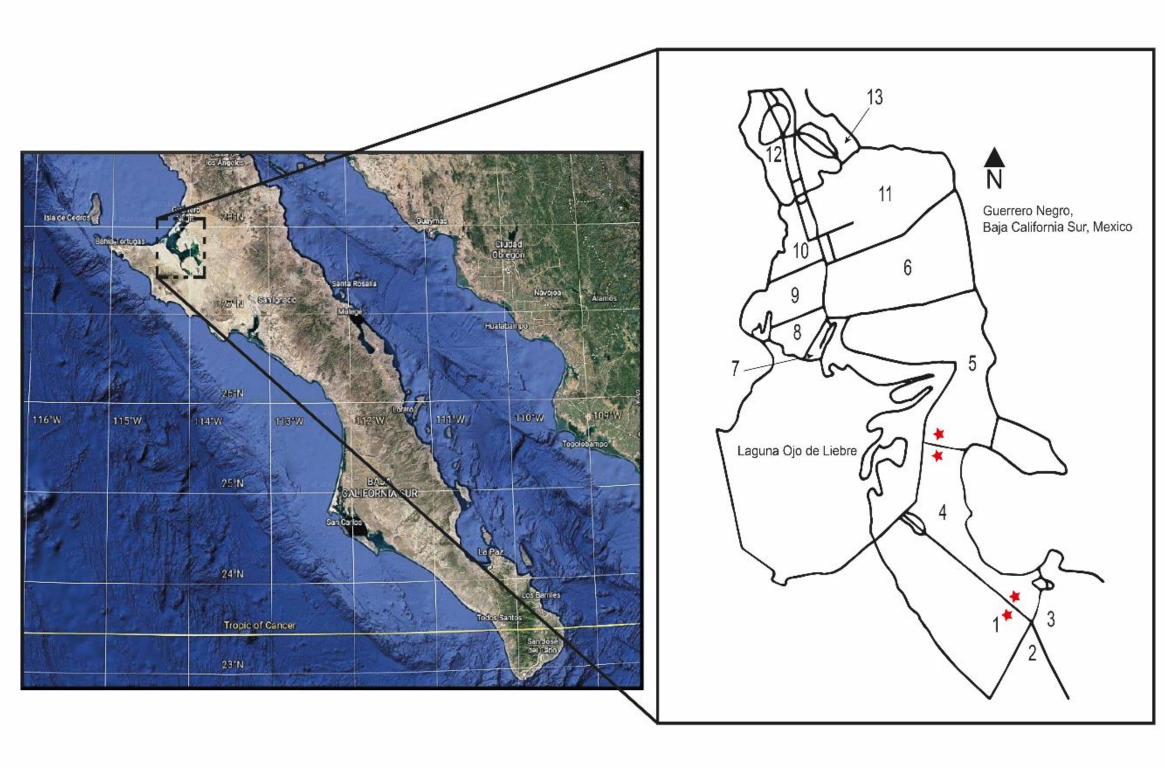

Figure S1. Map of the studied sites. At left, satellite map of Baja California Peninsula, photo property of Google Earth. At right, schematic representation of brine concentrators ponds (numbers) of Exportadora de Sal S.A. Sites where the microbial mats samples were collected are indicated with red stars.

Table S1. Sequences of primers used in the study.

Primer name	Targeted gene	5'-3' sequence	Tm °C	Expected amplicon size	Reference
515F-Y	*V4-V6 rRNA*	GTGYCAGCMGCCGCGGTAA	52	411	Parada et al. 2008
926R		CCGYCAATTYMTTTRAGTTT			
Illumina adapter + Primer 515-F-Y		TCGTCGGCAGCGTCAGATGTGTATAAGAGACAG + 515-F-Y	52	477	This study
Illumina adapter + Primer 926R		GTCTCGTGGGCTCGGAGATGTGTATAAGAGACA + 926R			
mlas-mod-F	*mcrA*	GGYGGTGTMGGDTTCACMCARTA	60-55	469bp	Angel et al. 2012
mcrA-rev-R		CGTTCATBGCGTAGTTVGGRTAGT			
mlas-mod-F + Illumina		TCGTCGGCAGCGTCAGATGTGTATAAGAGACAG + mlas-mod-F	60-55	536bp	This study
mcrA-rev-R + Illumina		GTCTCGTGGGCTCGGAGATGTGTATAAGAGACAG + mcrA-rev-R			

Table S2. Summary of 16S rRNA gene sequencing data from sampling sites after demultiplexing, denoising, and quality control of amplicons.

Sample	Raw data	Quality filtered	Denoised	Non-chimeric	Archaeal sequences	Bacterial sequences
A1a	16687	15998	15998	13868	557	13311
A1b	19417	18663	18663	15564	542	15022
A1c	19548	18728	18728	15692	208	15484
A4N1a	16841	16063	16063	14154	240	13914
A4N1b	16966	16127	16127	14407	224	14183
A4N1c	16461	15669	15669	14268	192	14076
A4N5a	14146	13649	13649	10765	40	10725
A4N5b	16052	15568	15568	12033	6	12027
A4N5c	16392	15906	15906	12784	22	12762
A5a	18164	17210	17210	14352	560	13792
A5b	16939	16136	16136	12384	303	12081
A5c	16011	15224	15224	11896	508	11388

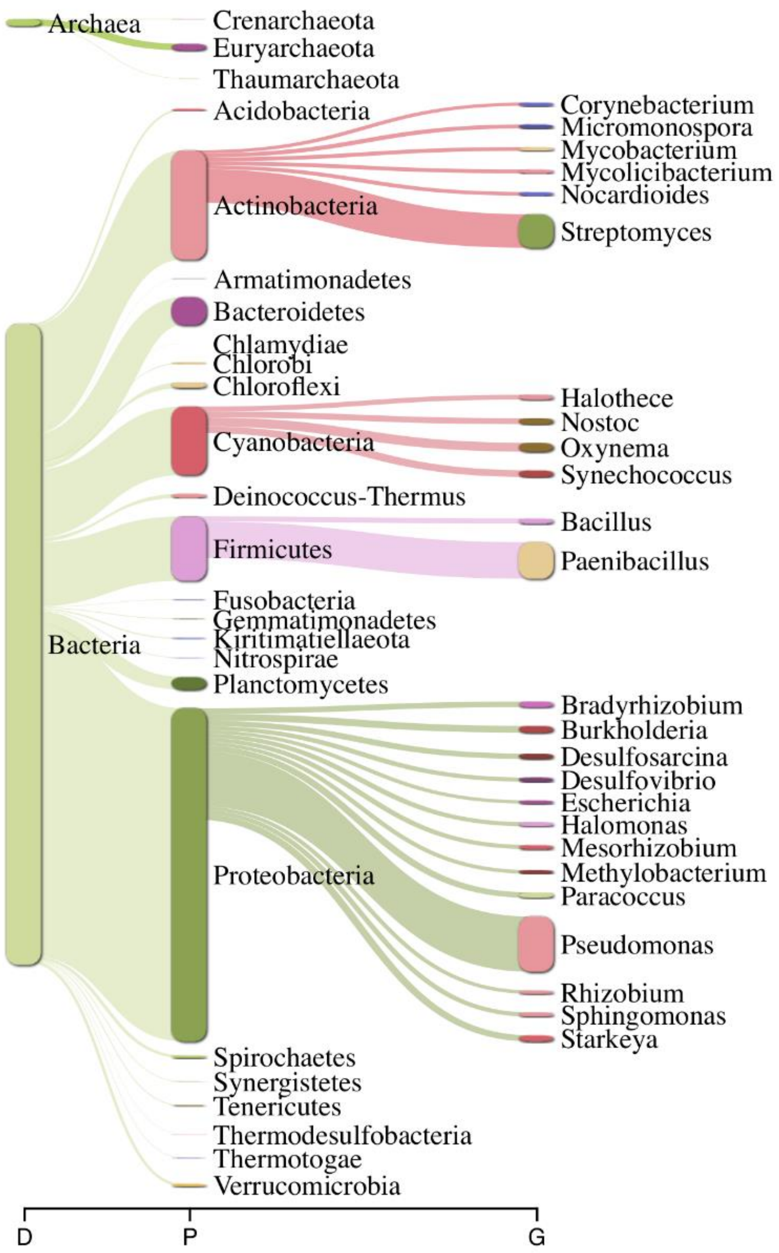

Figure S2. Sankey diagram showing the relative abundances of taxonomic groups at domain (D), phylum (P) and genus (G) ranks for whole metagenome shotgun reads of sample A5. Abundances were calculated using Kraken2 v. 2.1.2 and adjusted with Bracken v. 2.6.2 versus the k2_pluspf_8gb database downloaded on 2021-05-17. The diagram was visualised using Pavian v. 1.0 (10.1093/bioinformatics/btz715).

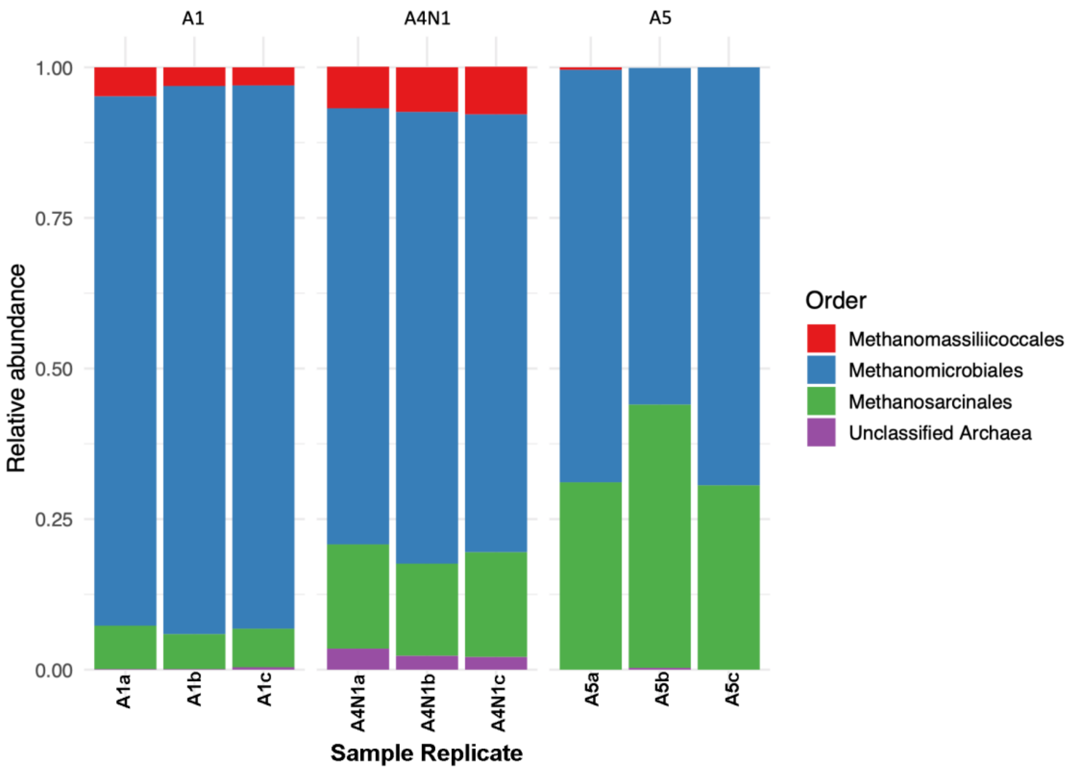

Figure S3. Taxonomic profile of *mcrA* amplicon reads rarefied to the smallest sample (8,299 sequences). Only groups containing > 10 reads are shown. Taxonomic labels were retrieved from the BLASTX best-hit versus Luke McKay's *mcrA* database (see methods for details).
